# The Effect of Breastfeeding Versus Sensorial Saturation on Infants’ Behavioral Responses of Pain following Pentavalent Vaccination on 4 and 6 Month Old Infants: A Randomized Controlled Clinical Trial Study

**DOI:** 10.30476/IJCBNM.2021.87090.1400

**Published:** 2022-04

**Authors:** Zohreh Karimi, Narges Kazemi Karani, Ebrahim Momeni, Ardashir Afrasiabifar

**Affiliations:** 1 Department of Operating Room, School of Paramedicine, Yasuj University of Medical Sciences, Yasuj, Iran; 2 Department of Nursing, School of Nursing, Yasuj University of Medical Sciences, Yasuj, Iran

**Keywords:** Breastfeeding, Pain, Infant

## Abstract

**Background:**

Pain is the fifth vital sign and its proper management in infants is important. The aim of this study was to compare the effect of breastfeeding versus sensorial saturation on infants’ behavioral responses of pain following Pentavalent vaccination on 4 and 6-month old babies.

**Methods:**

This single blind randomized controlled clinical trial study was conducted on Shahid Dastgheib Health center in Yasuj, from May to September 2016. Eligible infants (N=180) were selected using convenience sampling method. Then, using block randomization method, we assigned the participants to one of the three groups of Breastfeeding (N=60), Sensorial saturation (N=60), and Control (N=60). The infants’ behavioral responses of pain were measured using the Modified Behavioral Pain Scale (MBPS) and duration of crying following Pentavalent vaccination. Data were analyzed through SPSS software 22 using Chi square, Analysis of Variance (ANOVA), Kruskal-Wallis, and Dunn ’s multiple comparison tests. P<0.05 was considered statistically significant.

**Results:**

The results of the study showed that the MBPS mean scores for breast feeding, sensorial saturation, and control groups in 4-month old infants were 6.60±1.60, 5.40±1.30 and 8.90±0.40, and those of 6-month old ones were 7.20±1.10, 6.10±0.90 and 9±0.50, respectively. Also, both the breastfeeding and the sensorial saturation groups scored significantly lower in behavioral responses of pain and crying duration on both 4 and 6-month old infants compared to the control group (P<0.05). Sensorial saturation group significantly scored lower in behavioral responses of pain and crying duration on 4 and 6 month old infants than the breastfeeding group (P<0.05).

**Conclusion:**

Both breastfeeding and sensorial saturation could diminish the infants’ behavioral responses of pain following Pentavalent vaccination; however, sensorial saturation was more effective than breastfeeding.

**Trail Registration Number:**

IRCT2016051527916N1

## INTRODUCTION

Experience of pain has multidimensional aspects, including physiological, sensorial, emotional, cognitive, and behavioral aspects. ^1^ Vaccination is one of the common sources of pain resulting from receiving health care in children. ^2^ Pain, fear, and anxiety from vaccination may affect the acceptance of treatment negatively. Also, parents are not satisfied with the existing methods for pain relief, and this inadequate pain management causes negative experiences. ^3^ It was already believed that neonates and infants do not feel pain, but the results of some studies has shown that they even feel pain more than children and adults. They are also able to recall the painful events and show more severe backlash in repeating the painful procedures. ^4 , 5^ Infants respond to painful stimuli by physiological and behavioral changes. ^6^

Today, awareness of the consequences of untreated pain has resulted in additional efforts to diagnose, assess, and manage it. ^7^ In painful procedures, pain can be reduced by pharmacological and non-pharmacological methods. ^8^ A previous study showed that a low number of nurses use non-pharmacological methods in pain management for infants and children. ^9^ According to the results of studies, non-pharmaceutical interventions are effective and safe to reduce pain in children; thus, they have been considered more. ^10 , 11^

Distraction is a non-pharmacological method that can reduce pain and stressful behavior in children undergoing invasive treatment procedures. ^12^ Simultaneous stimulation of the senses of touch, smell, hearing, taste, and sight (sensorial saturation) during painful procedures enhances the pain-relieving effect of using a single technique. ^13 , 14^ Combination of stimuli, i.e. touch, hearing, smell, taste and sight, and maintaining eye contact with sucking and hugging increase the degree of impact. ^15^

Breast-feeding is another non-pharmacological method during painful procedures, such as vaccination, that reduces pain through skin contact between the mother and infant and creation of a sense of relief in infants through breast milk elements that block the neuronal routes in the spinal cord. ^16 - 20^

Due to the limited number of studies on measuring the palliative effect of sensorial saturation method on vaccination pain in infants and application of breastfeeding as a convenient, safe, non-invasive, cost-effective method that is accepted by mothers and health center staff; therefore, the present study aimed to compare the effect of breastfeeding versus sensorial saturation on the infants’ behavioral responses of pain following Pentavalent vaccination on 4 and 6-month old infants.

## MATERIALS AND METHODS

The study population of this single blind randomized controlled clinical trial consisted of at 4-month-old infants who referred to Shahid Dastgheib Health center for Pentavalent vaccination on Yasuj city, the south of Iran, during the period of May-September 2016. The sample size was estimated to be 60 infants in each group using the following formula and considering parameters of α=0.05, 1-α=0.95, $z_{1-\alpha /2}=1.96$ , β=0.2, 1-β=0.8, $z_{1-\beta }=0.85$ , effect size or d (µ_1_-µ_2_)=0.6, and standard deviation (S_1_=0.9, S_2_=0.7) based on the previous study. ^11^

$n=\frac{2[{\left(z_{1-\frac{\alpha }{2}}+z_{1-\beta }\right)}^{2}](s_{1}^{2}+s_{2}^{2})}{{\left({µ}_{1}-{µ}_{2}\right)}^{2}}=\frac{2[{\left(1.96+0.85\right)}^{2}]({\left(0.9\right)}^{2}+{\left(0.7\right)}^{2})}{{\left(0.6\right)}^{2}}=57$

The inclusion criteria consisted of healthy infants, no history of hospitalization and chronic disease, chronological age of 4 months old, no receive analgesic for 48 hours of pre-vaccination, babies exclusively fed with breast milk, not breast-fed for 30 minutes before vaccination, awake and calm infants (in this center, the oral polio vaccine is given at the age of 4 and 6 months, which is before the injection of the Pentavalent vaccine, and if the infants cry, they are first calmed and then the Pentavalent vaccine is injected(, babies with a dry diaper, and the parents’ consent to take part in the intervention. Exclusion criteria were lack of consent of the parents to continue cooperation in the project, diagnosis of a chronic disorder or history of hospitalization during the past 2 months between the two steps of the intervention, and occurrence of complications attributed to the pertussis vaccine after Pentavalent vaccination at 4 months of age.

**Figure 1:**
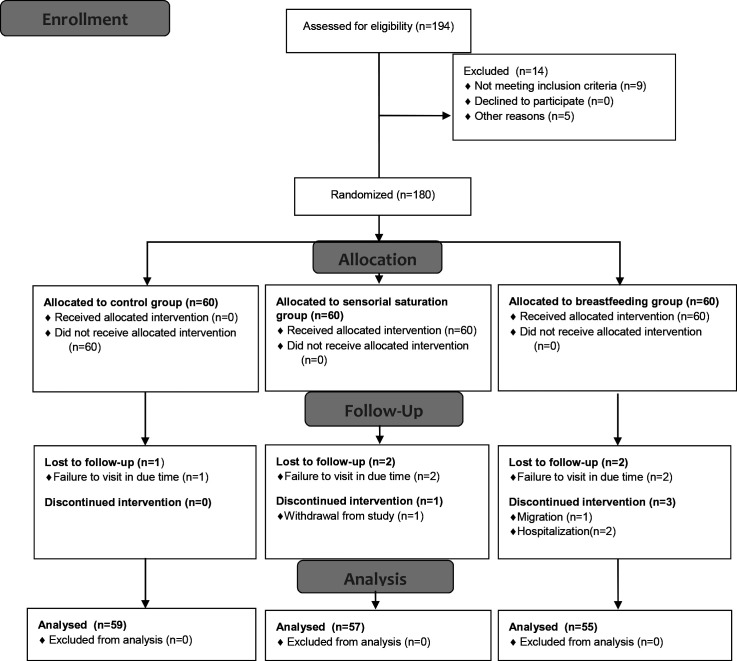
CONSORT flowchart of the participants

Infants (N=194) were initially assessed for eligibility; however, 14 infants were excluded and 180 infants were enrolled. Eligible infants were selected using convenience sampling method, according to block randomization; they were randomly assigned to one of the three groups of Breastfeeding (Group A=60 infants), Sensorial saturation (Group B=60 infants), and Control (Group C=60 infants). Since three groups participated in this study, we created six blocks, namely ABC, ACB, BAC, BCA, CAB, and CBA based on the factorial rule (3! = 3×2×1=6). There were three participants in each block. However, their arrangement varied. We selected the blocks from these six blocks, using replacement random sampling. All participants completed the study at 4-months of age, but 9 infants did not continue the interventions at 6 months of age. Therefore, we lost 9 infants at 6 months of age, and data of 171 infants, as Breast-feeding (N=55), Sensorial saturation (N=57) and Control group (N=59) were finally analyzed (Figure 1). 

The parent ʼs written informed consent was obtained and then they participated in the study. The study groups were as follows:

Breastfeeding: The infants’ mother was instructed to start her infant breastfeeding before vaccination and to continue it for two minutes; then, breastfeeding was discontinued and the vaccination was done.

Sensorial Saturation: This intervention was conducted by the second author. The researcher laid the infant on one side of the body, and the legs and arms were flexed without movement limitation. Only the movement of the vaccinated organ was limited. Then, 2^cc^ of Dextrose water solution 50% was slowly poured using a needleless syringe on the infant’s tongue. Two drops of Lavender extract (Zardband Company, confirmed by the Ministry of Health, Treatment and Medical Education of Iran, with patent No. 70,069,006,810,002 and factory standard ­of 65.81.00001) was poured to stimulate the infant’s olfactory sense, using a sterile gauze, and then it was put 30 cm ­from the infant’s head. At the same time, the infant’s attention was distracted by the researcher also through closely looking at the infant’s face, gently talking and constantly massaging the infant’s face and back. 

Control group: The infants were vaccinated according to the routine care of vaccine center. In all three groups, the infant was laid on a bed, and the vaccine was injected by a vaccinator. Then, the infant was returned to the mother’s cuddle, duration of crying was measured, and Modified Behavioral Pain Scale (MBPS) was completed. There were the same conditions such as ambient temperature, light, and sound, type and temperature of the injected solution for all groups. 0.5^CC^ Pentavalent vaccine was injected in the anterolateral half of the Vastus Lateralis muscle of the left side with Auto-disable syringe by the vaccinator who was a fixed person (health center staff).

After two months, at the time of vaccination at 6 months of age, vaccination was performed under the same conditions, the duration of crying was measured and recorded, and MBPS was completed.

Outcome variables of this study were the infants’ behavioral responses of pain and their crying duration following Pentavalent vaccine on 4 and 6 months of age. 

MBPS was used to measure the infants’ behavioral responses of pain following Pentavalent vaccination. Behavioral Pain Scale was firstly developed to assess pain in infants by Robieux and colleagues in 1991. Three subscales were defined: Facial expression, Cry, and Movements. Scores of Cry and Movements subscales ranged from 0-3, Facial Expression subscale from 0-2, and total score of scale ranged from 0-8. ^21^ Then, this tool was modified to assess immunization related pain in infants aged 2 to 6 months by Taddio and colleagues in 1995. They called it MBPS. Scoring of this scale was modified by them as Facial expression (0-3), Cry (0-4) and Movements (0-2-3) with total score of scale 0-10. Thus, higher scores mean worse infants’ behavioral responses of pain. The validity of the MBPS including Concurrent validity (r=0.74) using visual analogue scale, and Construct validity using two groups of infants who received the local anesthetic cream and placebo, was compared- was tested on 4 to 6 month-old infants for decreasing pain from diphtheria-pertussis-tetanus vaccine. They also assessed its reliability including Internal Consistency of items (r=0.67, r=0.48 and r=0.54), Inter-rater agreement (r=0.95), and test-retest reliability (r=0.95). ^22 , 23^

This scale has been used by Iranian researchers. For example, the validity and reliability of this tool has already been confirmed in the research of Taavoni et al. in 2009. They used simultaneous observation method for reliability, and correlation coefficient was reported (r=0.862) for 5 seconds before vaccine injection and (r=0.853) 15 seconds after injection. Content validity was checked by 10 faculty members of the Department of Midwifery, Nursing and Maternal Infants and Child Nursing. ^24^

In this study, duration of crying (per seconds) following Pentavalent vaccination was measured from the entrance of needle of vaccine into the infant’s body up to stopping his or her crying by the researcher. In this study, all evaluations were performed by a researcher.

Data were analyzed through SPSS software, version 22, using descriptive and inferential statistics, considering P<0.05. Chi-square and Analysis of Variance (ANOVA) tests were used for demographic variables including the infants’ sex and body weight. First, the distribution of the outcome variables (Facial expression, Cry, Movements, total MBPS or pain intensity, and duration of crying) was assessed using Kolmogorov-Smirnov test. Since their distribution was not normal, the results of non-parametric tests such as Kruskal-Wallis test and Dunn ’s multiple comparison test were reported for between group and pairwise comparisons, respectively. The data analyst was also blind to the type of interventions.

This study was approved by the Research Ethics Committee of Yasuj University of Medical Sciences with the ethics code of IR.YUMS.REC.1395.20. It was also registered in Iranian Registry of Clinical Trials. The voluntary participation and freedom to withdraw at each stage of the research were also emphasized.

## RESULTS

The result of the study showed that 79 (46.20%) infants were female and 92 (53.80%) were male. The means of weight of 4- and 6-month-old infants were reported to be 6.70±0.70 and 7.50±0.80 kg, respectively. No statistically significant difference was observed by sex and body weight in the three groups (P>0.05). 

The result of the study showed that the total MBPS or pain intensity mean scores for breast feeding, sensorial saturation, and the control groups in 4-month-old infants were 6.60±1.60 (Median=6), 5.40±1.30 (Median=6), and 8.90±0.40(Median=9), respectively. These values for 6-month-old ones were 7.20±1.10 (Median=7), 6.19±0.90 (Median=6), and 9±0.50 (Median=9), respectively. The infants in the breastfeeding group and the sensorial saturation group scored lower in behavioral responses of pain and crying duration following Pentavalent vaccine than those in the control group in both 4- and 6-month-old infants. Also, among group comparison using the Kruskal Wallis test indicated that the three groups significantly differed from each other in both 4-month-old (Table 1) and 6-month-old babies (Table 2) (P=0.001).

**Table 1:** Among group comparison for the infants ʼ behavioral responses of pain and their crying duration following Pentavalent vaccination in 4-month-old infants

Group Outcomes	Breastfeeding		Sensorial saturation		Control		P value*
	Median	IQR^b^	Median	IQR	Median	IQR	
Facial expression	2	1	2	1	3	0	0.001
Cry	2	1	2	0	3	0	0.001
Movements	2	0	2	0	3	0	0.001
Pain intensity (total MBPS^a^)	6	2	6	1	9	0	0.001
Duration of crying (s)	27	29	18	10	58	22	0.001

a Modified Behavioral Pain Scale,

b Interquartile Range, *Kruskal-Wallis test

**Table 2:** Among group comparison for the infants ʼbehavioral responses of pain and their crying duration following Pentavalent vaccination in 6-month-old infants

Group Outcomes	Breastfeeding		Sensorial saturation		Control		P value*
	Median	IQR^b^	Median	IQR	Median	IQR	
Facial expression	2	1	2	0	3	0	0.001
Cry	3	1	2	1	3	0	0.001
Movements	2	1	2	0	3	0	0.001
Pain intensity (total MBPS^a^)	7	2	6	1	9	0	0.001
Duration of crying (s)	35	27	23	10.50	75	40	0.001

a Modified Behavioral Pain Scale,

b Interquartile Range, *Kruskal-Wallis test

As previously mentioned in the subsection of data analysis, we performed Post HOC *using* Dunn ’s multiple comparison test considering Bonferroni Correction equal to 0.0167. First, in the two tests, the breastfeeding and sensorial saturation groups were compared with the control group in behavioral responses of pain and crying duration following Pentavalent vaccination on both 4- and 6-month-old subjects. The results showed that both the breastfeeding and sensorial saturation groups scored significantly (P<0.05) lower in behavioral responses of pain and crying duration following Pentavalent vaccination than the control group. 

Second, the breastfeeding group was compared with the sensorial saturation group. The results of this pairwise comparison indicated that the sensorial saturation group significantly (P<0.05) scored lower in behavioral responses of pain and crying duration following Pentavalent vaccination than the breastfeeding group. In other words, sensorial saturation was more effective than breastfeeding to diminish the infants’ behavioral responses of pain and crying following Pentavalent vaccination in both age groups (Table 3).

**Table 3:** Pairwise comparison for the infants’ behavioral responses of pain and their crying duration following Pentavalent vaccine in 4- and 6-month-old infants

Subscales	Dunn ’s Multiple comparison	4-month old			6-month old		
		Median	IQR^b^	P value*	Median	IQR	P value*
Facial expression	Breastfeeding – Control	3	1	<0.001	3	1	<0.001
	Sensorial saturation –Control	2.50	1	<0.001	3	1	<0.001
	Breastfeeding - Sensorial saturation	2	0	0.001	2	0	0.004
Cry	Breastfeeding – Control	3	1	<0.001	3	0	<0.001
	Sensorial saturation – Control	3	1	<0.001	3	1	<0.001
	Breastfeeding - Sensorial saturation	2	1	0.004	3	1	0.002
Movements	Breastfeeding – Control	3	1	<0.001	3	1	<0.001
	Sensorial saturation – Control	2	1	<0.001	2.50	1	<0.001
	Breastfeeding - Sensorial saturation	2	0	0.083	2	0	0.003
Pain intensity (total MBPS^a^)	Breastfeeding – Control	9	2.25	<0.001	9	2	<0.001
	Sensorial saturation – Control	8	3	<0.001	8	3	<0.001
	Breastfeeding - Sensorial saturation	6	1.75	0.004	7	1	0.001
Duration of crying (s)	Breastfeeding – Control	48	36.75	<0.001	50	43.75	<0.001
Sensorial saturation – Control	38	43	<0.001	41	52.75	<0.001
Breastfeeding - Sensorial saturation	20	20.75	0.001	24	19.50	0.009

a Modified Behavioral Pain Scale,

b Interquartile Range, *Dunn ’s multiple comparison test

## DISCUSSION

The results showed that both breastfeeding and sensorial saturation could diminish the infants’ behavioral responses of pain and crying duration following Pentavalent vaccination than infants in the control group in both age groups. In addition, sensorial saturation was more effective than breastfeeding in reducing the infants’ behavioral responses of pain and crying following Pentavalent vaccination on both ages.

Similar to our study results, a study in which the vaccine was injected while breastfeeding without interruption, breastfeeding was described as an effective way to reduce pain. ^25^ In another study, researchers assessed the infant pain during vaccination using the Face, Legs, Activity, Cry, Consolability pain scale. They reported that using breastfeeding significantly reduced the vaccination pain. ^26^ In another study, infants in the breastfeeding group and the combination of breastfeeding and music therapy had significantly shorter crying times, and the mean pain score during and one minute after heel blood sampling was lower compared to the control and music therapy groups. In this study, infants were breastfed for 5 minutes before heel blood sampling and the instrument used was Neonatal Infant Pain Scale. ^27^ In another study, researchers concluded that the mean pain intensity in the three groups of swaddling, breastfeeding and the combination of these two methods was significantly different from the control group. In this study, the infants were breastfed for 45 minutes before the vaccine and the data collection tool was Neonatal Facial Coding System. ^28^

Although the tools, age and method of breastfeeding in the above studies were different from the present study, in this study, the method of breastfeeding by the mother has been an effective method to reduce the pain of vaccination. In any case, breastfeeding reduces pain through distractions such as sucking, contacting the mother’s skin with the infant, creating a feeling of security and relaxation with the help of breast milk elements, and blocking the neuronal pathways in the spinal cord. ^29^

In a study, the babies were distracted by music, and in another study the infants were stimulated at the time of vaccination by using deep breathing with blowing paper whirligigs and deep breathing. In both studies, the infants’ pain scores at the time of vaccination were lower in the intervention group than in the control group. ^30 , 31^ Taste stimulation in the sensorial saturation group in the present study was done by dextrose. Sugar solutions are most commonly used to reduce the infant pain. In one study, the use of 25% glucose solution before immunization was able to reduce pain in healthy newborns. ^32^ In another study, the use of sucrose solution before vaccination significantly reduced the pain score and crying duration in the intervention group compared to the control group. ^33^ The results of the above studies are consistent with the sensorial saturation method in the present study.

Higher efficacy of the sensorial saturation method than breastfeeding method in the present study can be due to the fact that sensorial saturation, in addition to distraction, had the soothing effects of sweet solution and lavender odor. ^34 , 35^ In one study, both breastfeeding and seed therapy were effective in reducing vaccination pain and the efficacy of breastfeeding was significantly more than seed therapy. ^36^ However, in the present study, the efficacy of sensorial saturation method was greater on pain reduction than breastfeeding at the time of vaccination. This can be due to differences in the methods of breastfeeding between the two studies. In the above study, the vaccine was injected when the researcher observed active sucking and continued breast-feeding, while in the present study, the mother breastfed the infant and then slowly cut it after two minutes; then, vaccination was done on a bed. Seed treatment is effective in reducing pain with simultaneous application of two methods of distraction techniques and acupressure. 

In a previous study, glucose solution and lidocaine cream were both more effective than breast milk in reducing pain intensity and duration of crying in neonates. ^37^ In the mentioned study, 2 ml breast milk was given by syringe to the neonates that can justify lack of efficacy of breast milk, as in this study, the infants were fed directly from the mother’s breast. 

Promotion of breastfeeding, randomized allocation and the use of a control group are the strengths of this study. On the other hand, the present study suffered from limitations that should be considered to generalize its results. First, this study assessed the infants’ behavioral responses of pain following Pentavalent vaccination. We know that the pain related to vaccine is considered as transient pain and differs from acute and chronic pains. Second, we could not have within group comparisons due to a 2-month interval between our two measures.

## CONCLUSION

Both breastfeeding and sensorial saturation could diminish the infants’ behavioral responses of pain and crying duration following Pentavalent vaccination than infants in the control group in both ages. In addition, sensorial saturation was more effective than breastfeeding in diminishing the infants’ behavioral responses of pain and crying following Pentavalent vaccination in both age groups. Further investigations are recommended to confirm the results of this study. Both interventions were non-invasive, and they can simply be conducted before painful procedures.

By considering the above mentioned limitations, more investigations are recommended in order to reach better clinical judgments about the infants’ behavioral responses of pain in different conditions in terms of the type of pain or its duration. It is also suggested that the infants’ behavioral responses of pain should be examined using mixed designs of between- and within-subjects measures.

## ACKNOWLEDGEMENT

We thank all the mothers’ infants and the staff of Yasuj Shahid Dastgheib Health center and the Vice Chancellor for Research of Yasuj University of Medical Sciences who helped us to conduct this study with 23.2.808 grant number.

**Conflict of Interest:** None is declared. 
